# *Toxoplasma* Does Not Secrete the GRA16 and GRA24 Effectors Beyond the Parasitophorous Vacuole Membrane of Tissue Cysts

**DOI:** 10.3389/fcimb.2018.00366

**Published:** 2018-10-18

**Authors:** Shruthi Krishnamurthy, Jeroen P. J. Saeij

**Affiliations:** Department of Pathology, Microbiology and Immunology, School of Veterinary Medicine, University of California, Davis, Davis, CA, United States

**Keywords:** *Toxoplasma gondii*, secreted effectors, tissue cyst wall, tetracycline inducible expression, GRA16, GRA24

## Abstract

After invasion, *Toxoplasma* resides in a parasitophorous vacuole (PV) that is surrounded by the PV membrane (PVM). Once inside the PV, tachyzoites secrete dense granule proteins (GRAs) of which some, such as GRA16 and GRA24, are transported beyond the PVM likely *via* a putative translocon. However, once tachyzoites convert into bradyzoites within cysts, it is not known if secreted GRAs can traffic beyond the cyst wall membrane. We used the tetracycline inducible system to drive expression of HA epitope tagged GRA16 and GRA24 after inducing stage conversion and show that these proteins are not secreted beyond the cyst wall membrane. This suggests that secretion of GRA beyond the PVM is not important for the tissue cyst stage of *Toxoplasma*.

## Introduction

*Toxoplasma gondii,* which belongs to the phylum Apicomplexa, is an obligate intracellular parasite that can cause disease in immuno-compromised patients and fetuses (Montoya and Liesenfeld, 2004; Weiss and Dubey, 2009). It is the causative agent of toxoplasmosis, the 2nd most common cause of food-borne illness in the USA (Jones and Dubey, 2012). Infectious tissue cysts are present in brain and muscles of many warm-blooded chronically infected hosts (Kim and Weiss, 2004). Infected cats, which are the definitive hosts, shed infectious oocysts in their feces contaminating food and water sources. Infection is initiated by ingestion of either tissue cysts containing the bradyzoite life-cycle stage or oocysts (Dubey, 1998). Upon stage conversion into tachyzoites and invasion of a host cell, *Toxoplasma* forms a parasitophorous vacuole (PV) that is surrounded by the PV membrane (PVM) (Black and Boothroyd, 2000). During invasion, *Toxoplasma* secretes effector proteins from rhoptries (ROPs), which mediate invasion, inhibition of host restriction factors, and modulation of host signaling pathways (Dubremetz, 2007; Boothroyd and Dubremetz, 2008; Hakimi et al., 2017). Once inside the PV, proteins from the dense granule secretory organelles (GRAs) are secreted onto, and beyond the PVM into the host cell cytoplasm (Hakimi and Bougdour, 2015; Hakimi et al., 2017). Three putative translocon proteins: Myc-regulation 1 (MYR1) along with MYR2 and MYR3 determine transport of GRA16 and GRA24 across the PVM into the host cell cytoplasm after which they traffic to the host cell nucleus (Franco et al., 2016; Marino et al., 2018). In addition to these putative translocon proteins, an aspartyl protease, ASP5 cleaves many secreted GRA proteins at a characteristic RRLxx motif also known as the *Toxoplasma* export element (TEXEL) motif which is important for their localization and function (Coffey et al., 2015; Hammoudi et al., 2015). Most of these effectors that have been characterized are from the non-orally infectious tachyzoite stage. It is unclear if bradyzoites within tissue cysts, akin to tachyzoites within the PV, can secrete GRAs beyond the PVM as the cyst wall is built on the inside of the PVM (Jeffers et al., 2018) and presents a potential barrier for GRA secretion into the host cell.

GRA16, GRA24, GRA28 and IST (*T. gondii* inhibitor of STAT1 transcriptional activity) are secreted by tachyzoites beyond the PVM and traffic to the host nucleus (Bougdour et al., 2013; Braun et al., 2013; Gay et al., 2016; Nadipuram et al., 2016; Olias et al., 2016) where they modulate host signaling pathways important for parasite fitness. In this brief report we use the tetracycline inducible system to induce the expression of epitope tagged GRA16 and GRA24 after *in vitro* stage conversion. We observed that anhydrotetracycline (ATc) induced *GRA16*-HA and *GRA24*-HA are not secreted beyond the PVM and are not localized to the host cell nucleus. Instead, they accumulate within the *in vitro* tissue cysts.

## Materials and methods

### Host cells and parasite strain

Human foreskin fibroblasts (HFFs) were used as host cells and were cultured under standard conditions using Dulbecco Modified Eagle Medium (DMEM) with 10% fetal bovine serum (FBS) (Rosowski et al., 2011). We chose GT1 parasites expressing tetracycline repressor (Tet-R) (Etheridge et al., 2014), a type I strain that is capable of forming cysts during *in vitro* stage conversion (Lindsay et al., 1991; Fux et al., 2007) induced by pH 8-8.2+low CO_2_ (Skariah et al., 2010).

### Plasmid construction

Using Gibson assembly (Gibson et al., 2009), we constructed Tet-On plasmids with a phleomycin resistance cassette to express GRA16-HA and GRA24-HA. The pTeton vector backbone was amplified with the following primers which were used to construct both the pTetONGRA16-HA SRS22A3′UTR as well as the pTetONGRA24-HA SRS22A3UTR constructs:

vector TetON for-GCATCCACTAGTGCTCTTCAAGGTTTTACATCCGTTGCCT

Vector TetON rev-AATTGCGCCATTTTGACGGTGACGAAGCCACCTGAGGAAGAC

The following primers were used to amplify the pieces for pTetONGRA16-HA SRS22A3′UTR Gibson assembly:

Vector GRA16 for-ACCGTCAAAATGGCGCAATTATGTATCGAAACCACTCAGGGATAC

SRS22UTRGRA16 rev-AATGACAGGTTCAAGCATAATCGGGAACGTCGTATG

GRA16HA SRS22A for-TTATGCTTGAACCTGTCATTTACCTCCAGTAAACATG

SRS22Avector rev-TGAAGAGCACTAGTGGATGCGTTCTAGTGCTGTACGGAAAAGCAAC

The following primers were used to amplify the pieces for pTetONGRA24-HA SRS22A3′UTR Gibson assembly:

vectorGRA24 for –ACCGTCAAAATGGCGCAATTATGCTCCAGATGGCACGATATACCG

SRS22AUTRGRA24HA rev-AATGACAGGTTTAAGCATAATCGGGAACGTCGTATG

GRA24HASRS22A For-TTATGCTTAAACCTGTCATTTACCTCCAGTAAACATG.

### Parasite transfection and selection

The pTetOn vectors containing GRA16-HA and GRA24-HA were linearized using the AseI restriction enzyme. The linearized plasmids (50 μg) were electroporated into 5 × 10^7^ GT1tetR parasites using the protocol described in (Gold et al., 2015). After lysis of parasites from host cells, they were selected twice with 50 μg/ml phleomycin (Ble) and maintained in 5 μg/ml Ble (Krishnamurthy et al., 2016) after which they were cloned by limiting dilution.

### Indirect immunofluorescence with tachyzoites

Coverslips with a monolayer of HFFs were infected in the presence or absence of 2 μM ATc with GT1-TetR parasites or single clones of parasites expressing GRA16-HA and GRA24-HA under the RPS13 promoter downstream of a Tet-O7 operator. The coverslips were fixed 16 h post infection with 3% formaldehyde and processed for IFA using rabbit anti-HA primary (Roche) antibody followed by goat anti-rabbit Alexa-555 secondary antibody and Hoechst (Invitrogen).

### *In vitro* stage differentiation and IFA with tissue cysts

HFFs were infected with a single clone of GT1-tetR parasites that expressed GRA16-HA only in the presence of ATc. The media was switched from DMEM with 10% FBS to tricine-buffered RPMI media with pH 8.0 and put in an incubator with low CO_2_ after 24 h of infection (MOI = 0.1) to induce tachyzoite to bradyzoite stage conversion. After 5 days, 2 μM ATc was added and 2 days later the coverslips were fixed with 100% cold methanol (also eliminates YFP signal from TetR) and processed for IFA. The cyst wall was stained with DBA-FITC (Boothroyd et al., 1997) along with HA and Hoechst as described above.

## Results

To test if GRAs are secreted beyond the tissue cyst wall membrane, we utilized the Tet-inducible system (Etheridge et al., 2014) to express an HA-tagged copy of GRA16 or GRA24 under the Tet operator (Tet-O) in parasites expressing Tet-R. We could not just use *GRA16*-HA or *GRA24*-HA expressed from the endogenous promoter because if we saw these proteins in the host nucleus we would not know if they were secreted beyond the PVM before the cyst wall was made (as tachyzoites) or after the cyst wall was made (as bradyzoites). In the absence of anhydrotetracycline (ATc), the Tet-R binds to Tet-O and represses the transcription of either *GRA16*-HA or *GRA24*-HA under the RPS13 promoter (Etheridge et al., 2014; Wang et al., 2016). ATc binds TetR and relieves repression of transcription which allows for the expression of HA-tagged GRA16 or GRA24. Since ATc is smaller than the size-exclusion limit of the cyst wall (Lemgruber et al., 2011), we decided to use this system to answer our question.

**Figure 1:**
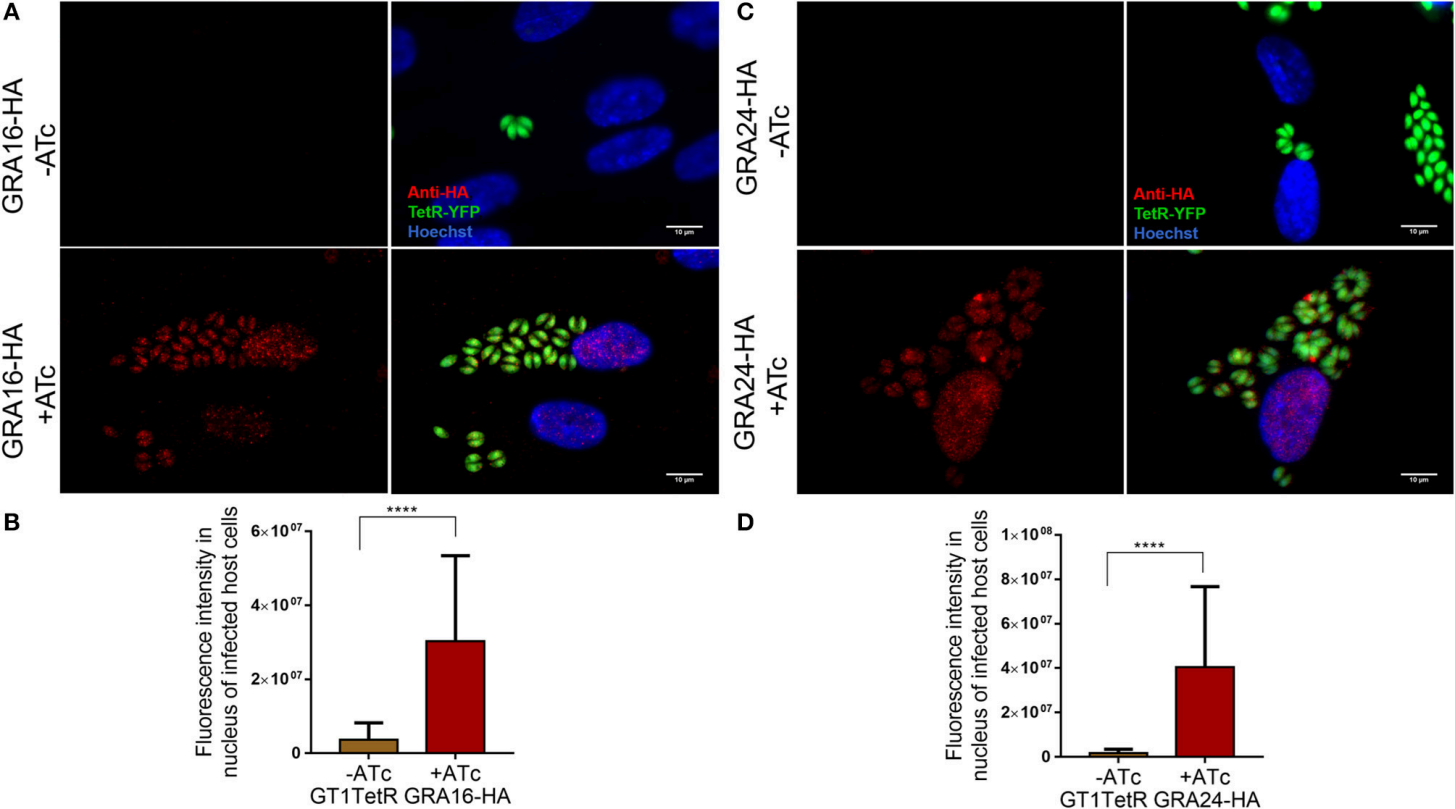
GT1- TetR parasites express GRA16-HA and GRA24-HA only in the presence of ATc. A monolayer of HFFs were infected with single clones of GT1 Tet-R parasites, which contained either GRA16-HA or GRA24-HA cloned in front of a TetOn promoter, in the presence or absence of ATc. Cover slips were fixed 16 h post infection and processed for IFA as described. Tet-R is YFP tagged and GT1 Tet-R parasites do not express any proteins that are HA-epitope tagged (first panel). In the absence of ATc, Tet-R represses the expression of GRA16-HA (**A** first panel) and GRA24-HA (**C** first panel). Repression by Tet-R is relieved only in the presence of 2 μM ATc (second panel) allowing for the proper localization of GRA16-HA **(A)** and GRA24-HA **(B)** to the parasite PVM and host cell nucleus. Images are scaled to 10 μm. Quantification of GRA16-HA **(C)** and GRA24-HA **(D)** secreted into the host cell nucleus only in presence of ATc was done using ImageJ. Error bars indicate mean with SD and significance was determined by performing Student's *t*-test from 3 biological replicates using Graph Pad Prism7.0 (****P < 0.0001).

To check if our constructs were able to stably express functional GRA16 and GRA24, we first transfected them into the RH parasite strain and observed nuclear localization of these proteins (data not shown). After transfection of the GT1 Tet-R expressing strain with the Tet-inducible GRA16-HA or GRA24-HA construct and subsequent selection with phleomycin for stable integration, we show by IFA that in tachyzoites GRA16-HA (Figures 1A,B) and GRA24-HA (Figures 1C,D) were only expressed in the presence of ATc.

**Figure 2:**
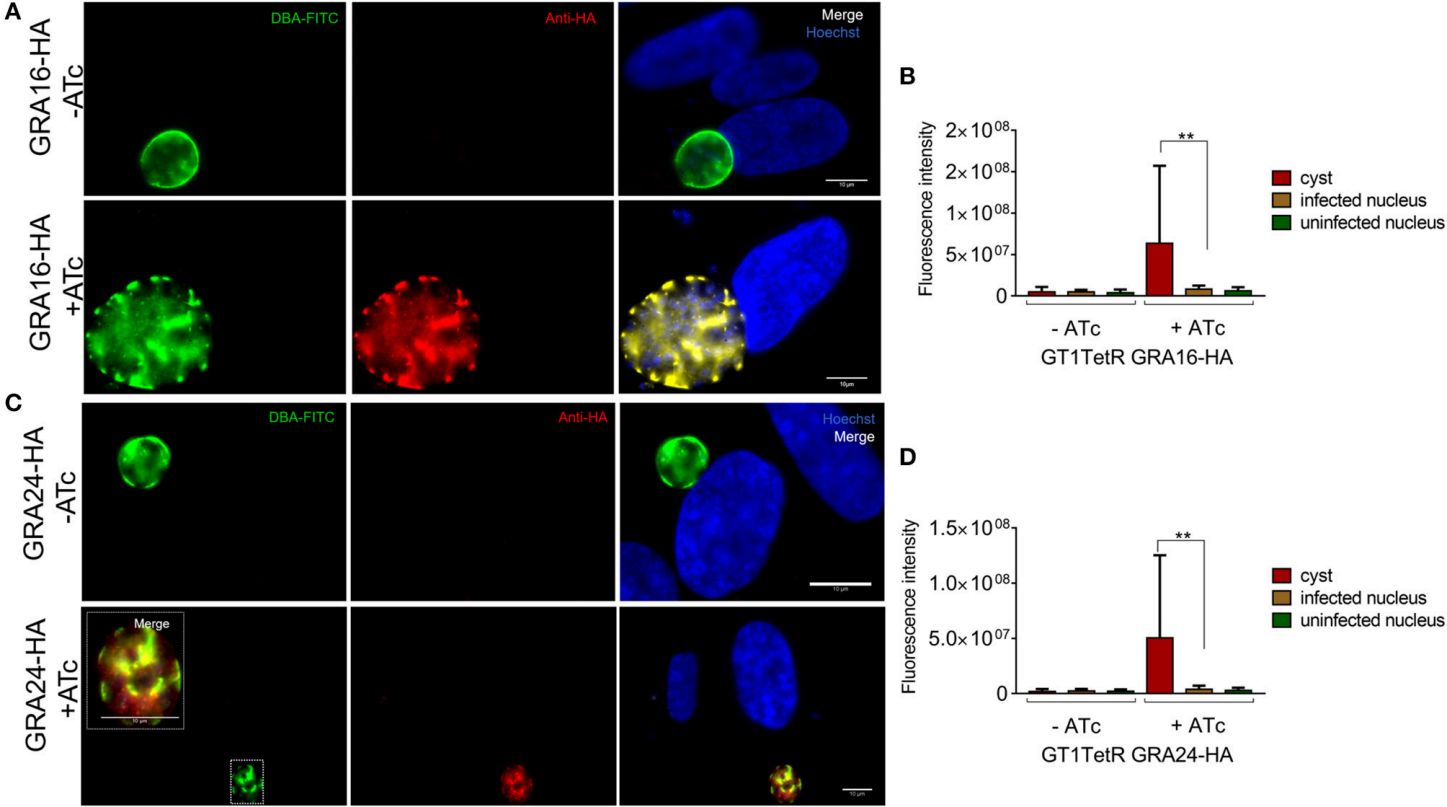
*In vitro* DBA positive cysts do not secrete GRA16-HA and GRA24-HA beyond the cyst wall membrane. Stage differentiation was induced using single clones of parasites, which contained either GRA16-HA or GRA24-HA cloned in front of a TetOn promoter, 16 h post infection of coverslips containing HFFs. ATc was added to the monolayer containing cysts 5 days after inducing stage differentiation. Coverslips were processed for IFA with DBA-FITC to stain the cyst wall and anti-HA antibody to track the localization of GRAs16 and 24. In the absence of ATc, GRA16-HA (**A** first panel) and GRA24-HA (**A** first panel) were not detected in parasites. GRA16-HA (**A** second panel) and GRA24-HA (**B** second panel, inset) accumulated beneath the cyst wall only in the presence of ATc. Images are scaled to 10 μm. Fluorescence intensity was measured to quantify the localization of GRA16-HA and GRA24-HA in the nucleus of infected cells, uninfected cells as well as the tissue cysts in the presence and absence of ATc **(C,D)**. Signal intensity was the highest and above background (uninfected cells) only for cysts in the presence of ATc. Error bars indicate mean with SD and significance was determined using Student's *t*-test from 3 biological replicates (**P < 0.05).

A single parasite clone that expressed GRA16-HA or GRA24-HA only in the presence of ATc was chosen for induction of stage differentiation *in vitro* in human foreskin fibroblast (HFFs). Five days post-switching, 2 μM of ATc was added to the cultures to induce the expression of GRA16-HA and GRA24-HA since we observed that at least 50% of the parasites had converted to cysts by staining the cyst wall with DBA-lectin (Boothroyd et al., 1997) (data not shown). The parasites were fixed 48 h following addition of ATc to allow for sufficient expression of GRA16-HA and GRA24-HA. We performed an indirect immunofluorescence assay (IFA) to determine the localization of GRA16 and GRA24 using anti-HA antibody as well as DBA-lectin to detect the cyst wall. We show that in host cells containing tissue cysts, GRA16-HA and GRA24-HA were not detected in the host cell nucleus or beyond the tissue cyst wall membrane and that instead they accumulated underneath the cyst wall. Almost 100% of vacuoles we observed were DBA positive. We decided to observe HFFs only infected with one parasite and therefore containing only one cyst as differences in the timing of conversion could affect the localization of GRA16 and GRA24. Out of 189 (80 for GRA16-HA and 109 for GRA24-HA from three biological replicates) images of singly infected host cells containing DBA positive cysts, both GRA16 and GRA24 were expressed exclusively beneath the cyst wall only in the presence of ATc (Figures 2A,B). We observed GRA16 and GRA24 localized to the host cell nucleus only in multiple infected cells containing tachyzoites, along with *in vitro* cysts (Supplemental Figure 1). Thus, our results show that GRA16 and GRA24 are not secreted beyond the tissue cyst membrane into the host cell.

## Discussion

We show here that GT1 parasites are able to form DBA lectin positive *in vitro* cysts. We also show for the first time that ATc is able to cross the cyst wall *in vitro*. Even though bradyzoites within tissue cysts are not as metabolically active compared to tachyzoites, it is becoming clear that they are also not in a dormant state (Sinai et al., 2016). However, we observed that bradyzoites do not secrete GRA16 and GRA24 beyond the *in vitro* cyst wall membrane. These proteins accumulated within the cyst wall suggesting that their role in the host cell nucleus is not required at this stage. In preparing for the chronic phase of their life cycle, bradyzoites lose connectivity between themselves through the intravacuolar network (IVN) and undergo asynchronous division (Frénal et al., 2017). Our data indicates that the bradyzoites within cysts also lose connectivity to host cells by not secreting GRAs, which usually modulate host signaling pathways in tachyzoites, beyond the PVM. Possibly bradyzoites require these proteins during natural oral infections after excystation from tissue cysts to establish infection in gut epithelial cells of the host. Not secreting parasite proteins beyond the cyst wall might help *Toxoplasma* to remain invisible and undetected by the host immune response during the chronic phase of infection. This hypothesis is in conjunction with published literature wherein proteins from dense granules (Ferguson, 2004; Lemgruber et al., 2011) and rhoptries (Schwarz et al., 2005) were shown to be secreted in bradyzoites but never beyond the tissue cyst wall. Another possibility may be that the translocon proteins MYR1/2/3 or ASP5 are not sufficiently expressed at this stage to effectively mediate transport of secreted GRAs beyond the cyst wall membrane. Even though all the MYRs and ASP5 are expressed in tachyzoites, sporozoites and bradyzoites, their expression is significantly lower in bradyzoites (Marino et al., 2018). However, even if MYR1-3 and ASP5 are expressed, our data indicate that the cyst wall seems to act as a barrier as ATc- induced GRA16 and GRA24 accumulated beneath the wall (Figure 2).

## Author contributions

SK generated all the data. SK and JS wrote and edited the manuscript.

### Conflict of interest statement

The authors declare that the research was conducted in the absence of any commercial or financial relationships that could be construed as a potential conflict of interest.

## Supplementary material

Supplemental Figure 1 Localization of GRA16-HA and GRA24-HA to the host cell nucleus was observed only in multiple infected host cells containing parasites in different stages of *in vitro* stage conversion. Parasites expressing GRA16-HA **(A)** or GRA24-HA **(B)** localized the respective epitope tagged GRAs in the parasites as well as in the host cell nucleus. Non-uniform DBA staining suggests that the parasites were in different stages of conversion into tissue cysts. Images are scaled to 10 μm.

## Abbreviations

Tet-on: tetracycline inducible system
TetR: tetracycline repressor protein
ATc: anhydrotetracycline
HA: hemagglutinin epitope tag
YFP: yellow fluorescent protein
Ble: phleomycin
HFF: human foreskin fibroblast
DBA lectin: dolichos biflorus agglutinin
IFA: indirect immunofluorescence
GRA: dense granule proteins
ROP: rhoptry proteins
IST: *T. gondii* inhibitor of STAT1 transcriptional activity
PV: parasitophorous vacuole
PVM: PV membrane.
