# Supplementary figures and images for: Toxoplasma Does Not Secrete the GRA16 and GRA24 Effectors Beyond the Parasitophorous Vacuole Membrane of Tissue Cysts
Source: Front Cell Infect Microbiol. 2018 Oct 18;8:366. doi: 10.3389/fcimb.2018.00366 (PMC6201044; doi:10.3389/fcimb.2018.00366)

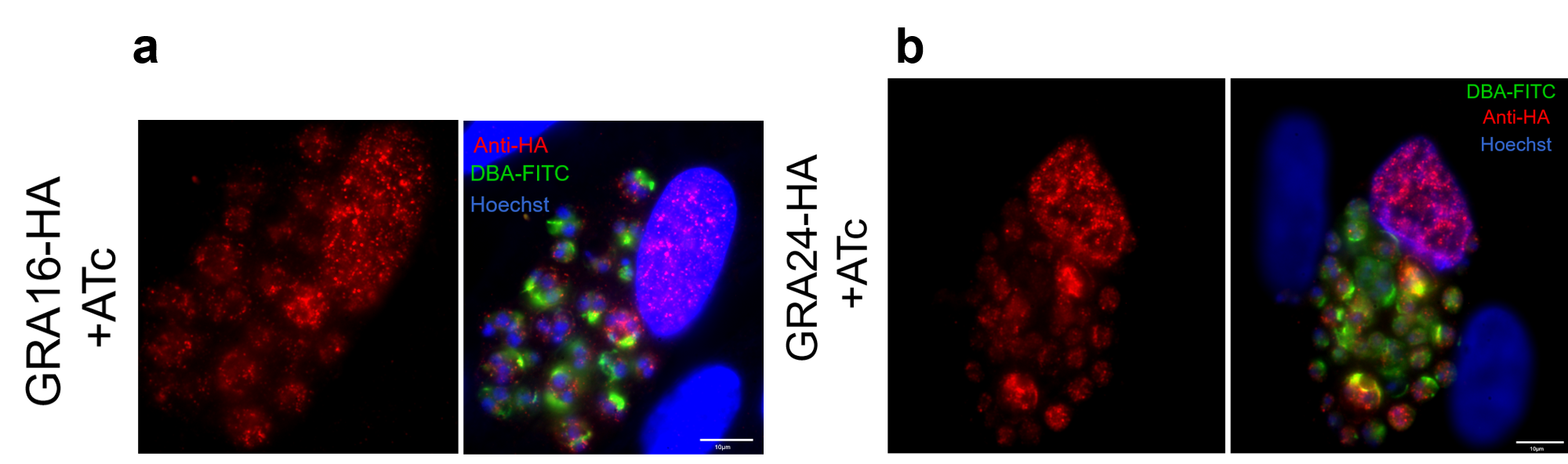

Supplement: Supplemental Figure 1 — Localization of GRA16-HA and GRA24-HA to the host cell nucleus was observed only in multiple infected host cells containing parasites in different stages of in vitro stage conversion. Parasites expressing GRA16-HA (A) or GRA24-HA (B) localized the respective epitope tagged GRAs in the parasites as well as in the host cell nucleus. Non-uniform DBA staining suggests that the parasites were in different stages of conversion into tissue cysts. Images are scaled to 10 μm. [file Image_1.TIF]
